# The impact on dietary outcomes of licensed and brand equity characters in marketing unhealthy foods to children: A systematic review and meta‐analysis

**DOI:** 10.1111/obr.13443

**Published:** 2022-03-09

**Authors:** Jessica Packer, Simon J. Russell, Katie McLaren, Gabriela Siovolgyi, Claire Stansfield, Russell M. Viner, Helen Croker

**Affiliations:** ^1^ Population, Policy and Practice Research and Teaching Department, UCL Great Ormond Street Institute of Child Health University College London London UK; ^2^ EPPI‐Centre, UCL Social Research institute University College London London UK

**Keywords:** child and adolescent health, food marketing, obesity, policy research

## Abstract

Licensed and brand equity characters are used to target children in the marketing of products high in fat, salt, and sugar (HFSS), but the impact of characters on dietary outcomes is unclear. The primary aim of this review was to quantify the impact of both licensed and brand equity characters on children's dietary outcomes given that existing regulations often differentiates between these character types. We systematically searched eight interdisciplinary databases and included studies from 2009 onwards until August 2021, including all countries and languages. Participants were children under 16 years, exposure was marketing for HFSS product with a character, and the outcomes were dietary consumption, preference, or purchasing behaviors of HFSS products. Data allowed for meta‐analysis of taste preferences. A total of 16 articles (including 20 studies) met the inclusion criteria, of which five were included in the meta‐analysis. Under experimental conditions, the use of characters on HFSS packaging compared with HFSS packaging with no character was found to result in significantly higher taste preference for HFSS products (standardized mean difference on a 5‐point scale 0.273; *p* < 0.001). Narrative findings supported this, with studies reporting impact of both character types on product preferences including food liking and snack choice. There was limited evidence on the impact on purchase behaviors and consumption. These findings are supportive of policies that limit the exposure of HFSS food marketing using characters to children.

**Abbreviations**
CI: confidence Interval
EU: European Union
HFSS: high in fat, salt, and sugar
PRISMA: Preferred Reporting Items for Systematic Reviews and Meta‐Analyses
PROSPERO: The International Prospective Register of Systematic Reviews
SES: socioeconomic status
UK: United Kingdom
UNICEF: United Nations Children's Fund
US: United States
WHO: World Health Organization

## 1 INTRODUCTION

Children are exposed and targeted by a multitude of food and drinks marketing, the majority for products high in fat, salt, and sugar (HFSS).^1^, ^2^, ^3^, ^4^ Marketing increases short‐term consumption, preference for, and purchase intention of HFSS products by children.^5^, ^6^, ^7^ The impact on consumption can accumulate, as small excesses in daily energy intake can contribute to overweight and obesity over time.^8^ Marketing of HFSS food and drinks is high on the public health and policy agenda; the use of characters in marketing has been identified as a persuasive technique by the World Health Organization (WHO)‐UNICEF‐Lancet Commission.^9^ Marketing includes any form of commercial communication that acts to advertise or promote a product or service.^10^ It occurs across a plethora of mediums, including television (TV), films, radio, magazines, digital mediums (social media platforms, websites, apps, streaming services, advergames, and text/email), physical mediums (billboards, point‐of‐purchase displays), and sport sponsorship. WHO has recommended reducing the marketing of HFSS/unhealthy foods and nonalcoholic beverages to children, but this is complex due to the variety of mediums across which marketing occurs.^10^

The importance of including both brand equity and licensed characters within restrictions is specifically stated by the WHO,^11^ as advertising restrictions commonly differentiate between licensed characters (borrowed with no brand association, e.g., Disney characters) and brand equity characters (created by the brand, e.g., Coco the Monkey). Several countries include the use of characters in their restrictions (e.g., the United Kingdom, Ireland, Australia, Chile, and Portugal), but this commonly only applies to licensed characters.^12^, ^13^, ^14^, ^15^ Brand equity characters are often exempt from HFSS marketing restrictions (e.g., the United Kingdom, Ireland, and the voluntary EU pledge from leading food and beverage companies) and are allowed to “sell the products they were designed to sell.”^14^, ^16^ Despite the WHO recommending that packaging be considered a form of marketing, it is not included in restrictions in most countries.^12^, ^13^, ^14^, ^15^ Restrictions also commonly apply only to predigital media and therefore need to be updated in line with the shift to digital marketing.^14^, ^17^ In 2020, nearly all children in the United Kingdom aged 5–15 went online, and over half use social media apps/sites.^18^ Chile is an exception, where comprehensive policies exist that ban the use of characters across all HFSS product marketing.^19^

Content analyses and systematic reviews of marketing practices reveal characters as one of the most commonly used tactics for promoting food and drinks, disproportionately HFSS products, to children on packaging^20^, ^21^ and TV.^22^, ^23^ There is some evidence that licensed and brand equity characters increase children's food preference, choices, intake, and purchasing behaviors in relation to HFSS products.^6^, ^24^, ^25^ However, there are limitations in the current reviews; one did not provide the search strategy and therefore was not replicable,^24^ a lack of focus on characters,^6^, ^25^ and effects not being quantified through meta‐analysis. Therefore, the current literature does not provide information on the specific impact of characters on child dietary outcomes.

We undertook a systematic review to synthesize the most up‐to‐date evidence on how characters used in marketing, specifically advertising and packaging, impact a range of children's diet‐related outcomes. We aimed to extend previous work by quantifying the effects of characters in marketing on children's purchasing behaviors and intentions; children's food preference and objectively measured food consumption (e.g., energy intake/quantity of items consumed); and examining whether character type influenced the response. We also sought to assess if child characteristics (e.g., age, socioeconomic status [SES]) or marketing format (content within advertisements vs. on packaging) impacted responses. This work is of particular relevance for informing policy makers and in formulating evidence‐based advertising regulations.

## 2 METHODOLOGY

### 2.1 Protocol and registration

The current systematic review was registered with PROSPERO (registration number: CRD42019153853) and conducted and reported using the PRISMA checklist.^26^

### 2.2 Eligibility criteria, information resources, and search strategy

Quantitative peer‐reviewed articles/studies (experimental with appropriate comparison group or nonexperimental “real world”) were eligible for inclusion; population criteria were children and adolescents (aged between 0 and 16 years, in line with UK advertising regulation definition of children); all geography, languages, and studies between 2009 and August 2021 were included; intervention criteria were any form of HFSS food advertisement featuring a character (i.e., TV, packaging, and advergames); and outcomes were HFSS food consumption (objectively measured, that is, ad libitum consumption), food preferences (i.e., self‐reported and like/dislike ratings), and food purchasing behavior or intention (i.e., quantity of product purchased and intention to purchase or to ask/pester parent to purchase). Studies from 2009 onwards were included to ensure results were most relevant to current marketing strategies and advertising practices. Exclusion criteria were participant age (over 16 years), date of publication (pre‐2009), design (content analysis, reviews, and qualitative or nonpeer review, e.g., dissertations), intervention (no HFSS advertisement exposure with a character), and outcome measure (no measure of food choice, consumption, intake, purchase, and purchase/pester intention).

Searches were conducted on October 22, 2019, and updated on August 16, 2021. The following databases were searched: MEDLINE (Ovid), Cochrane Library, Scopus, PsycInfo (Ovid), ProQuest (Central)—ASSIA, Web of Science—Social Science Citation Index and Emerging Sources Citation Index, and Social Policy and Practice (see Table S1 for details of search and Table S2 for search history). Search results were firstly imported into EndNoteX9 to remove duplicates and then into EPPI‐Reviewer 4, which was used for screening and to manage the search.

### 2.3 Study selection

Articles were double screened on title and abstract and then full text, with discrepancies jointly reconciled. Full‐text articles were acquired through web and online library services; all papers eligible for full‐text screening were retrieved successfully.

### 2.4 Data extraction and items

Data from included studies were independently doubly extracted. Additional data from six studies for meta‐analyses were requested from corresponding authors, of whom three responded with the required data.

Data extraction included study identification (authors, country, and year of publication), target population (children and/or adolescents), sample group description (size of sample, age range, and mean age of participants), study description (study design and assignment to conditions), intervention description (advertising medium and brand character), comparison type (HFSS food advertisement vs. healthy food or nonfood advertisement), test foods used, outcome type (consumption, preference, or purchasing), and outcome measures (kcal, kJ, grams, preference ratings, and purchase request measures).

### 2.5 Assessment of quality

The Cochrane Risk of Bias Tool (RoB 2)^27^ was used to assess included experimental studies for bias. This was undertaken doubly and independently, with discrepancies jointly reconciled.

### 2.6 Data synthesis

We completed descriptive synthesis of studies first and then explored potential for meta‐analysis. To be included in meta‐analysis, experimental studies were required to have an exposure of HFSS marketing that featured a character and a suitable comparison group, including HFSS marketing with no character, or healthy/nonfood marketing with or without a character. Due to the differences in the design (between‐subjects and within‐subjects), HFSS food product (cereal, crisps, etc.), and packaging exposure, a DerSimonian–Laird random‐effects model was used. We graphically presented the results using forest plots. Analyses were conducted using Stata (16.1, StataCorp LLC, College Station, TX).^28^ Subgroup analysis by character type was conducted, assessing the differential effects of licensed characters to brand equity characters. Sensitivity analysis was conducted by omitting studies assessed to have a high risk of bias, to test the stability of the results. Heterogeneity of studies was tested using the *I* ^2^ statistic, with a score >50 indicating presence of heterogeneity.^29^ Publication bias was assessed using Egger's test, funnel plot, and trim‐and‐fill analysis.^30^, ^31^

## 3 RESULTS

### 3.1 Study selection

**FIGURE 1:**
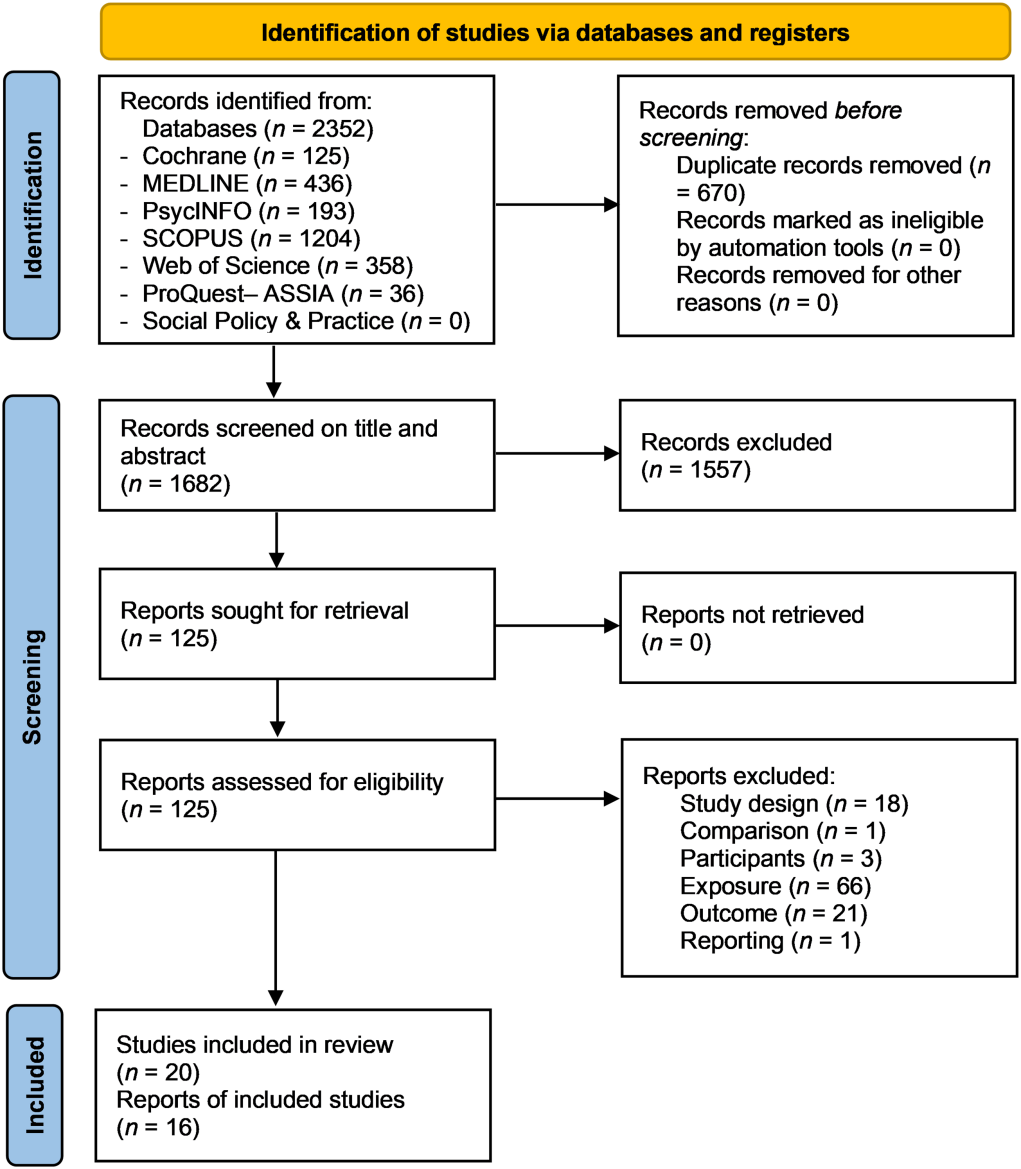
PRISMA screening flowchart

The database searches yielded 2352 articles, resulting in 1682 after removing duplicates. Following title and abstract screening, 1557 articles were excluded, and 125 were included to be screened on full text. This led to the final inclusion of 16 articles (20 studies), with five studies included in meta‐analysis (Figure 1). There were 109 articles excluded after screening on full text: 66 due to lacking an exposure that met all inclusion criteria (a HFSS product marketing that explicitly included a character), 21 did not have the outcome of interest, and 18 lacked a control or appropriate comparison group.

### 3.2 Study description and results

A summary of study information is provided in Table 1 including details on participants, setting, design, intervention, outcomes assessed, and findings.

**TABLE 1:** Descriptive table of character experimental studies

Author, country, year	Participants	Design	Advertising exposure	Comparison	Outcome	Relevant results	Risk of bias
Ares et al.,^32^ 2016, Uruguay	*N* = 239 (rating outcome, *N* = 120; choice outcome, *N* = 119) Age range = 6–12 Mean age = not stated	Experimental (school), within‐subject, position of labels randomized	Product label for HFSS foods (sponge cake and yogurt, fictitious brand) with unknown brand equity characters (bear and dinosaur)	Product label for the same HFSS products with no character	Preference: Product liking on a 7‐point scale (1, *I would not like it at all*; 7, *I would love it*) and forced pair label choice (“which of the 2 [product] would you like the most?”)	Selection of HFSS product with character on label was significantly greater compared with label with no character, regardless of age. Food product rating was not significantly different between labels with or without a character.	Low
Harris et al.,^33^ 2012, United States (Study 2)	*N* = 152 Age range = 7–12 Mean age = 9.4	Experimental (research left), between‐subject, random assignment	Advergames with HFSS products (Pop‐Tarts and Oreo cookies) and brand equity character (Pop‐Tart characters) or unknown brand equity characters (Oreo advergame), 12‐min playing time	Advergame with healthy products (fruit and vegetables) or nonfood advergame (Jewel Quest, Tumblebugs), 12‐min playing time	Consumption: Ad libitum snack consumption of HFSS products (crisps and cookies, fruit snacks and goldfish crackers) (grams) and healthy (grapes and carrots), 20 min	HFSS product consumption was significantly greater in HFSS advergame condition, compared with healthy advergame, and no significant difference compared with nonfood advergame.	Low
Jain et al.,^34^ 2011, India	*N* = 378 Age range = 13–17 Mean age = not stated	Experimental (school), between‐subject, allocation not specified	5‐ to 10‐min viewing of print advert of HFSS product (chocolate, fictitious brand) with licensed character (Mickey Mouse)	5‐ to 10‐min viewing of print advertisements of the same HFSS product with no endorsement	Purchase: Postintervention, purchase intention product (scale NS)	Purchase intentions of HFSS product endorsed by a licensed character were no different to the no‐endorsement control group.	Some concerns
Kotler et al.,^35^ 2012, United States	*N* = 343 (*N* = 207 subgroup) Age range = 2–6 Mean age = 4	Experimental (research left), between‐subject, random assignment	HFSS food image (crisps, chocolate, and donut—unbranded) with licensed character (Sesame Street) or unknown bran equity character sticker	HFSS or healthy food image with no character or healthy food image (fruit and vegetables) with character sticker	Preference: Forced preference choice (mix of food products and character conditions) Consumption: Consumption of food (pieces) in subgroup of participants (time not stated)	Preference was significantly greater in licensed character exposure, compared with unknown character and no character. Consumption increased with licensed characters compared with no character, significance unclear.	Low
Lapierre et al.,^36^ 2011, United States	*N* = 80 Age range = 4–6 Mean age = 5.6	Experimental (shopping left), between‐subject, random assignment	Packaging of HFSS cereal (fictitious brand “Sugar Bits”) with licensed character (Happy Feet)	The same HFSS cereal packaging with no character	Preference: Taste rating on a 5‐point Likert scale (smiley face)	Taste rating was significantly higher in character packaging condition compared with no character.	Some concerns
Letona et al.,^37^ 2014, Guatemala	*N* = 121 Age range = 4.3–11.5 Mean age = 7.4	Experimental (school), within‐subject, random order	Packaging of HFSS product (honey graham crackers and crisps, unbranded) with licensed characters (SpongeBob SquarePants, the Pink Panther, and El Chavo)	Packaging of the same HFSS product with no character	Preference: Taste rating on a 5‐point Likert scale (smiley face) of HFSS products (honey graham crackers and crisps)	Taste rating of HFSS products was significantly higher in character packaging condition compared with no character packaging.	Low
Leonard et al.,^38^ 2019, United States (Study 1a)	*N* = 26 Age range = 4–10 Mean age = 6.6	Experimental (public spaces, e.g., libraries, soccer games, and museums), within‐subject, exposure counterbalanced	HFSS snack (fruit flavored gummy snacks, Market Pantry) with a licensed character sticker on the packaged (SpongeBob SquarePants character)	The same HFSS product with no character sticker on package	Preference: Paired snack choice—children could choose from tray	Children were significantly more likely to choose the HFSS product with the licensed character, compared with product without licensed character.	High
Leonard et al.,^38^ 2019, United States (Study 1b)	*N* = 26 Age range = 4–10 Mean age = 6.6	Experimental (public spaces e.g., libraries, soccer games, museums), within‐subject, exposure counterbalanced	HFSS snack (fruit flavored gummy snacks, Welch's) in a package designed with an licensed character (Scooby‐Doo)	The same HFSS product with no character sticker on package and images of fruit	Preference: Paired snack choice—children could choose from tray	Children were significantly more likely to choose the HFSS product with the licensed character, compared with product without licensed character.	High
Leonard et al.,^38^ 2019, United States (Study 2)	*N* = 139 Age range = 3–12 Mean age = 7.4	Experimental (public spaces, e.g., libraries, soccer games, and museums), within‐subject, exposure counterbalanced	HFSS product (gummy snacks, brand not stated) with licensed character on package (Minions)	The same HFSS product without LC or healthy product (raisins, brand not stated) without LC	Preference: Paired snack choice—children could choose from tray	HFSS product with licensed character was significantly more likely to be selected than HFSS or healthy product without licensed character.	High
Leonard et al.,^38^ 2019, United States (Study 3)	*N* = 130 Age range = 4–7 Mean age = 5.6	Experimental (public spaces, e.g., libraries, soccer games, and museums), between‐subject, randomization not stated	HFSS product (cookies, fictitious brand, Snackcookies) with licensed character on package (Scooby‐Doo)	The same HFSS product without LC character on package (Scooby‐Doo)	Preference: Food liking on a 5‐point scale using emoticons, averaged score of taste rating (“Hate It” to “Love It”) and wanting rating (“How much would you like to have this food for a snack?” “Do not Want!” to “Want It!”) Consumption: Consumption **–** Ad libitum intake of cookies or dried apricots in bowls, dependent on condition (3 min)	No significant effect of licensed character compared with no character on HFSS consumption or food liking.	High
McGale et al.,^39^ 2016, United Kingdom (Study 1)	*N* = 60 Age range = 4–8 Mean age = 6.9	Experimental (school), within‐subject, random order	Packaging of HFSS product (Cheesestrings, Pom‐Bear Potato Snacks, and Coco Pops Snack Bar) with their brand equity characters (Coco the Monkey, Pom‐Bear, and Mr Strings)	Packaging of the same HFSS products with no characters	Preference: Taste rating on a 5‐point Likert scale (smiley face); paired snack choice and final snack choice out of the three products chosen from paired task	Mean taste rating of all products was significantly higher in character packaging condition, compared with no character. Final snack choice was significantly higher for food item with a brand equity character than no character food item, and a trend to select character option was seen for pair snack choice.	Low
McGale et al.,^39^ 2016, United Kingdom, (Study 2)	*N* = 149 Age range = 4–8 Mean age = 6.9	Experimental (school), within‐subject, random order	Packaging of HFSS products (Cheesestrings, Pom‐Bear Potato Snacks, and Coco Pops Snack Bar) with incongruent brand equity character (brand equity character of another product)	Packaging of the same HFSS products with no characters	Preference: Taste rating on a 5‐point Likert scale (smiley face); paired snack choice and final snack choice out of the three products chosen from paired task	Mean taste rating of all products was significantly higher in character packaging condition, compared with no character. No difference in final snack choice between conditions. Paired choice was significantly higher for food item with incongruent brand equity character than no character food item.	Low
Naderer et al.,^40^ 2017, Austria	*N* = 363 Age range = 6–15 Mean age = 10.55	Experimental (school), between‐subject, random assignment	Product placement of HFSS product (M&Ms) in movie (7‐min clip of Smurfs) ‐static placement condition (shown in background) ‐character product involvement (character interacts with the product)	Control, 7‐min clip of Smurfs with no product placement	Preference: Snack choice (M&Ms or 2 other chocolate snack options)	Both placement conditions were significantly more likely to pick the featured HFSS snack (M&Ms), compared with control and character product involvement condition significantly more likely than static placement condition. Age was unrelated to snack choice.	Some concerns
Ogle et al.,^41^ 2017, United States	*N* = 149 Age range = 6–9 Mean age = 7.36	Experimental (laboratory), within‐subject, random order	Packaging of HFSS product (dried fruit, bread, corn chips, yoghurt, and cereal—unbranded) with a licensed character (Lightning McQueen, Sponge Bob SquarePants, and Dora the Explorer)	Packaging of the same HFSS product with no character or more healthful product with and without character	Preference: Forced product choice between pairs, of which food they would want to eat	Less healthful products without characters were chosen significantly more than products with characters.	Low
Ponce‐Blandón et al.,^42^ 2020, Spain	*N* = 421 Age range = 4–6 Mean age = 4.8	Experimental (education lefts), between‐subject, random assignment	8‐min episode of cartoon (Caillou) with an advert for HFSS product (Bollycao chocolate‐filled roll) with licensed character (The Simpsons)	Control groups—no ad (Group 1) and nonfood ads (Group 4)	Preference: choice between two products—advertised product (Bollycao—chocolate‐filled roll) versus similar nonadvertised product (Qé Tentación—chocolate‐filled roll)	Children exposed to the advert with the licensed character were significantly more likely to prefer the advertised product compared with control.	Low
Putnam et al.,^43^ 2018, United States	*N* = 132 Age range = 4–5 Mean age = 4.8	Experimental (childcare facility), between‐subject, random assignment	Advergame with HFSS products (crisps or soft drink, assume unbranded) with licensed character (Dora the Explorer)	Advergame with healthy products (banana and orange juice) and the same licensed character or nonfood advergame with no character	Preference: Snack and drink product choice (snack: banana OR 28 g Lay's crisps; drink: 227 ml orange juice OR 213 ml can of soft drink—Coca‐Cola)	No significant differences in product choice by advergame condition (HFSS, healthy or nonfood).	Low
Rifon et al.,^44^ 2014, United States	*N* = 276 Age range = 5–10 Mean age = 7.3	Experimental (laboratory), between‐subject, random assignment	Advergame with HFSS product (cereal, unfamiliar brand, Honey O's) with unknown brand equity character, either integrated or in background and children assigned to play or watch	Advergame with no food and the same unknown character, children assigned to play or watch	Preference: Perceived taste rating on a 5‐point scale (stars) Purchase: Purchase request of HFSS product on a 5‐point Likert scale (thumbs)	Purchase request and taste expectations significantly greater in HFSS advergame conditions with characters (integrated: play/watch and background: play), compared with control. No significant difference if children watched the advergame with character in background.	Low
Roberto et al.,^45^ 2010, United States	*N* = 40 Age range = 3.8–6.2 Mean age = 5.0	Experimental (school), within‐subject, random order	Packaging of HFSS products (graham cracker and gummy fruit snacks, unbranded) with a licensed character (Scooby‐Doo, Dora the Explorer, and Shrek)	Packaging of the same HFSS products without character or healthy product (carrot) with and without character	Preference: Product preference and hypothetical snack choice between matched pair; taste rating on 5‐point Likert scale (smiley face)	Product preference, taste rating, and snack choice of HFSS products with characters on packaging were significantly greater than product packaging with no characters.	Low
Smith et al.,^46^ 2020, Australia	*N* = 156 Age range = 7–12 Mean age = 8.7 ± 1.5	Experimental study (university), between‐subject, random assignment	4‐min online advergame that promoted an HFSS product (gummy confectionery, unfamiliar brand) with an unknown brand equity character (avatar named Ziggy)	Control group with no advertising	Preference: Snack choice—between four options: test brand, supermarket brand, grapes or unknown brand Consumption: Consumption and energy intake (g, kcal) weight in grams of the chosen snack before and after 10‐min consumption time	Across groups, there were no significant differences between pregame and postgame ratings of taste or fun. Children in the advergame condition chose the advertised brand more frequently than control, but this was not found to be significant. There were no significant differences in consumption or energy intake between advergame and control.	Low
Smits and Vandebosch,^47^ 2012, Belgium	*N* = 57 Age range = 6–7 Mean age = 6.8	Experimental (school), between and within‐subject, random assignment and order	Images of HFSS food products (chocolate and cookies, unbranded) with picture of a licensed character (“Kabouter Plop”) or an unknown brand equity character (garden gnome—Kabouter Karel)	Images of the same food products with no character (baseline)	Consumption: Intended consumption frequency on a 10‐point scale (*never* to *every day*) Purchase: Intended frequency of purchase requests on the same scale	Intended consumption frequency and intended purchase request frequency were both significantly greater with either character compared with baseline and significantly greater with licensed character compared with unknown character. Trend for unhealthy products to have a greater intended purchase request.	Some concerns

### 3.3 Description of studies

The age of participants ranged from 2 to 15 years old; for the five studies included for meta‐analysis, participants' ages ranged from 4 to 11.5 years (mean = 6.5 years). The majority of studies were conducted in the United States (*n* = 11), followed by the United Kingdom (*n* = 2), India (*n* = 1), Australia (*n* = 1), Austria (*n* = 1), Belgium (*n* = 1), Guatemala (*n* = 1), Spain (*n* = 1), and Uruguay (*n* = 1). Studies tended to be experimental with a mixture of between‐subject and within‐subject designs, and most were conducted in schools (*n* = 9). The most common HFSS exposure was packaging with a character (*n* = 12), followed by advergames (*n* = 4), print adverts (*n* = 2), TV adverts (*n* = 1), or film product placement (*n* = 1). The featured characters included licensed (*n* = 12; including Dora the Explore and Scooby‐Doo), brand equity (*n* = 5; including Coco the Monkey and Pom‐Bear), or both (*n* = 3). HFSS products were a mix of familiar branded (e.g., Pom‐Bear Potato Snacks and Oreos), unfamiliar branded (i.e., only available in another country, Honey O's), fictitious brand (i.e., created for the experiment, Snackcookies and Sugarbits) or unbranded (i.e., shown in clear or no packaging; crisps and donuts). Outcomes, related to the marketed HFSS product, were preference (*n* = 17, including taste rating), purchasing behaviors (*n* = 3, including intention to purchase), and consumption (*n* = 5, including ad libitum intake).

### 3.4 Preference outcomes

The use of characters had a significant impact on HFSS product preference across most studies. Ten studies showed that the marketing of HFSS products with characters led to significantly higher preferences (including taste rating,^44^, ^45^ food liking,^32^ forced pair product choice,^32^, ^35^, ^39^, ^42^, ^45^ and snack choice^38^, ^39^, ^40^, ^45^) for the marketed HFSS products compared with the control conditions (no character with the same HFSS marketing or nonfood exposure). Of the other three studies, one compared characters in an HFSS advergame to a healthy advergame with character and a nonfood advergame with no character, with no significant differences found in forced snack choice between the three advergame conditions.^43^ A further study compared an HFSS advergame with a control condition and found children in the advergame condition chose the advertised snack choice more frequently than control, but this was not significant.^46^ Conversely, one study found that packaging without characters was chosen significantly more compared with packaging with licensed characters in a forced choice task.^41^

### 3.5 Meta‐analysis of studies examining taste preference

**FIGURE 2:**
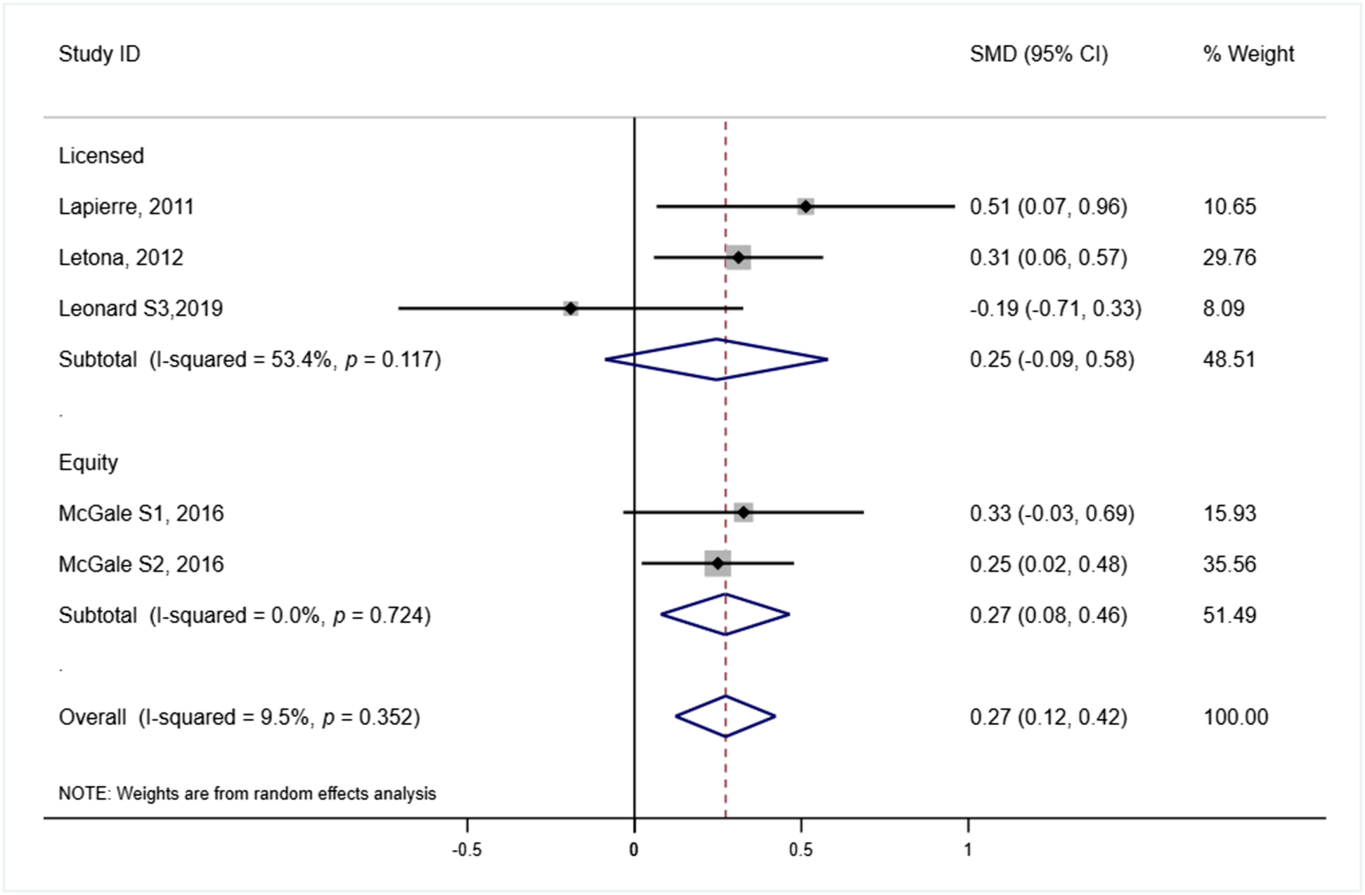
Forest plot showing standardized mean difference in taste preference of HFSS products between HFSS packaging with and without a character HFSS advert, by character type

Five studies provided sufficient data to be included in meta‐analysis of taste preference, three licensed and two brand equity.^36^, ^37^, ^39^ Taste preference or food liking (combined taste preference and food wanting) was measured on 5‐point smiley face Likert scales (1, *low preference*; 5, *high preference*), so standardized mean difference (SMD) was used. Further details of the outcome measures and the comparison groups are included in Table S3. The results from the meta‐analysis show that the use of a character in HFSS product packaging, compared with HFSS product packaging with no characters, resulted in a significantly higher taste preference for HFSS products with a pooled effect size of 0.273 (95% CI 0.123, 0.423; *p* < 0.001; *I* ^2^ = 9.5%; Figure 2). Subgroup analysis by character type found a significant effect for brand equity characters compared with no character (higher HFSS preference SMD = 0.272; 95% CI 0.079, 0.464; *p* = 0.006; *I* ^2^ = 0.0%); however, this was not significant for licensed characters (SMD = 0.245; 95% CI −0.088, 0.579; *p* = 0.150; *I* ^2^ = 53.4%). Egger's regression analysis showed no evidence of publication bias (*p* = 0.700), and trim‐and‐fill analysis suggested potential evidence of one missing study (see Figure S4). A sensitivity analysis was conducted, omitting the high risk of bias study,^38^ and was not found to impact the results (see Figure S5).

### 3.6 Purchase intentions

The use of characters had a significant impact on purchasing behaviors, with two studies finding evidence suggesting that the use of characters in HFSS marketing led to higher levels of intended requests of the marketed HFSS products compared with nonfood advergame^44^ or baseline for printed advert.^47^ One study found that there was no significant difference in purchase intentions for HFSS product between exposure to print advert with licensed character to no‐endorsement exposure.^34^

### 3.7 Consumption outcomes

Five studies measured consumption outcomes and found mixed evidence. Two studies found use of characters had a significant impact on consumption outcomes, with significantly higher ad libitum consumption of marketed HFSS products in an HFSS advergame condition compared with a healthy food advergame^33^; and intended consumption frequency of the marketed HFSS food was significantly higher following exposure to character endorsement compared with baseline, for both licensed and brand equity characters.^47^ Two studies showed no significant difference between conditions with and without licensed characters, on HFSS packaging^38^ or advergame,^46^ and results were unclear in another study.^35^

### 3.8 Impact of age

Evidence was mixed regarding how age influenced preference and consumption outcomes. Fifteen studies reported the impact of age; 10 found it was not a significant factor (including one consumption study),^3^, ^33^, ^38^, ^39^, ^40^, ^42^, ^45^, ^46^ one reported a directional effect/trend with age,^38^ one reported a trend for younger participants to be impacted more for preference outcomes,^32^ and three found that younger age significantly impacted preference outcomes (across 5–10, 6–9, and 4–6 age range, respectively).^37^, ^41^, ^44^

### 3.9 Other secondary outcomes

Three studies reported the impact of SES, and all found that SES had no impact on taste rating outcomes, but meta‐analysis by SES was not possible.^3^, ^39^ No studies reported whether advert format (e.g., TV advert vs. advergame) influenced outcomes or examined long‐term effects. Effect of character type was directly assessed in two studies; licensed characters resulted in significantly greater consumption and intended purchase requests^47^ and product preference,^35^ compared with brand equity characters.

### 3.10 Impact of country

Because a large proportion of studies were conducted in the United States (*n* = 11), the impact of country of origin for studies on general findings was considered. There was no difference in the results by country of study, with 7 out of 9 non‐US studies and 9 out of 11 US studies finding similar things.

### 3.11 Quality of studies

The risk of bias was assessed as mostly low, with three studies assessed as having some concerns and four studies, from the same article, assessed as high risk (see Figure S6 for bias assessment). The studies that were assessed as having some concerns were due to concerns with the randomization process^47^ or selection of reported result.^36^ The studies included in meta‐analysis were assessed as mostly low risk and sensitivity analysis was conducted to assess results when high‐risk study was omitted (see Figure S5).

## 4 DISCUSSION

Our extensive systematic review included the first meta‐analysis examining the impact of characters used in the marketing of HFSS products to children and found consistent evidence that marketing HFSS foods with characters influences children's preferences.

We found consistent evidence that HFSS packaging using both licensed and brand equity characters resulted in children having significantly higher preferences for an advertised product, compared with no character. The meta‐analysis results showed that the presence of a character resulted in a significantly higher taste preference for HFSS products, compared with packaging with no characters. The SMD of 0.27 indicates a relatively small effect^48^; however, small effects can accrue to create meaningful change at a population level.^49^ Characters appeared to positively impact purchase intentions, but evidence was more limited, and the evidence for consumption outcomes was mixed.

We found limited evidence that age and SES influenced the impact of advertising. There was some evidence of greater impact among younger compared with older children but no evidence of differences by sociodemographic circumstance. A recent review found consistent evidence from a large number of studies that children from lower SES and ethnic minority backgrounds are exposed to a greater amount of advertising for unhealthy products compared with less disadvantaged and nonethnic minority children.^50^ This suggests that, regardless of impact from a single advert exposure, children from minority and disadvantaged backgrounds are likely to be disproportionately affected by advertising and highlights the role of greater regulation in addressing health inequalities.

Our findings provide evidence that the use of characters, in particular brand equity characters, are effective marketing tactics that impact diet‐related outcomes in children. This is important to consider when developing policies aiming to reduce the impact of marketing on children's health. Children are uniquely susceptible to the effects of advertising, particularly because they are not cognitively mature and may not fully understand the intent of advertising.^51^ Characters are an influential tool in marketing as they appear to attract the attention and gain the trust of children, leading to increased brand recognition, positive brand attitudes, and brand loyalty.^52^ This is especially true for established characters, with whom children may form parasocial relationships (single‐sided connections between media users and on‐screen characters).^53^ This connection is exploited in marketing through evaluative conditioning, where positive feelings towards licensed or well‐known brand equity characters are used to transfer positive feelings to the marketed product.^54^, ^55^ This effect is used advantageously by brands through cross‐promotion, whereby licensed characters from popular children's media are utilized in HFSS marketing, and subsequent exposure to these characters in their original media (e.g., seeing licensed characters in movies and TV shows). This cross‐promotion creates additional marketing outside advertising contexts and beyond regulation.^52^, ^56^, ^57^ This suggests that the effects of using characters in marketing may be underestimated.

Characters are used extensively to market HFSS products to children.^20^, ^21^, ^23^, ^24^, ^25^ Potential areas for extending regulations include expanding broadcast regulations beyond child‐specific media, to nonbroadcast media. Our review included marketing of all types and provided evidence that nonbroadcast media, such as advergames and film product placement, are effective at influencing children's dietary behaviors. A prewatershed ban on HFSS adverts (between 5:30 a.m. and 9 p.m.) on TV and on demand program services, and a restriction on paid‐for‐less healthy food and drink advertising online, has been announced by the UK Government,^58^ which could overcome some of the gaps in regulation and lower exposure to character‐based HFSS advertising. The extension of broadcast regulations to cover product packaging has also been recommended,^21^ as the use of characters on packaging that targets children is unrestricted in the United Kingdom and elsewhere^13^, ^14^ and is pervasive on HFSS products.^21^ The impact of characters packaging on dietary outcomes is evidenced in our meta‐analysis and wider review. Some companies have voluntarily committed to limit the use of characters on packaging for some of their products, including cereal boxes at Lidl^59^ and products that do not meet specified thresholds at Unilever.^60^ This shows some awareness and recognition of the power of this marketing approach from the food and beverage industry, although self‐regulation of industry has typically not been effective and adherence to voluntary codes may not sufficiently reduce exposure.^61^, ^62^ Independent third party monitoring, with clearly defined and rigorous consequences, was recommended in a recent study on compliance and effectiveness of industry self‐regulation of HFSS food TV advertisement to children.^63^ Regulation and compliance across all potential marketing mediums is complex and challenging, but comprehensive restrictions and enforcement are likely to be necessary to effectively reduce children's exposure to marketing for HFSS foods.

### 4.1 Limitations

Our review has some limitations; only five studies were eligible for meta‐analysis, and the samples for character subgroup analysis were small; therefore, care due to variability needs to be taken. Although most were of reasonable quality with low/some concerns of bias, one study was deemed as high risk of bias as it was not randomized. Heterogeneity was low, and a random‐effects model was used to account for differences in the advertising exposures. The criteria of only including papers from 2009 onwards restricted the results to some extent but ensured that the findings were reflective of contemporary marketing practices and relevant to inform the thinking of policy makers. Due to a lack of studies measuring consumption and purchase behaviors, with appropriate comparisons, meta‐analysis was only possible for preference outcomes; five studies (from four articles) were included in meta‐analysis. A limitation to our secondary aim to explore outcomes by SES was that only three studies provided appropriate data or examined the impact of SES. A further limitation is that all included studies were experimental, despite inclusion criteria including real‐world studies; therefore, there would be various assumptions involved in extrapolating these findings to broader populations. Further primary research, especially for digital marketing and on social media platforms, would be useful in further developing the evidence base.

### 4.2 Conclusion

Our data provide further evidence that HFSS marketing using characters increases preference for HFSS products compared with not using characters and suggests purchasing behaviors and consumption are also deleteriously impacted. These findings suggest that reduced exposure of children to HFSS marketing including all character types and including packaging may have beneficial impacts upon dietary taste preferences and choices.

## CONFLICT OF INTEREST

The authors declare no conflict of interest.

## Supporting information




**Table S1:** Details of search
**Table S2:** Search history
**Table S3:** Rationale for meta‐analysis inclusion and data processing
**Figure S4:** Trim and fill analysis
**Figure S5:** Sensitivity analysis of meta‐analysis excluding Leonard study (high risk): Forest plot showing standardised mean difference in taste preference of HFSS products between HFSS packaging with and without a character HFSS advert, by character type
**Figure S6:** Bias assessment for experimental studies
