# Supplementary material for: The impact on dietary outcomes of licensed and brand equity characters in marketing unhealthy foods to children: A systematic review and meta‐analysis
Source: Obes Rev. 2022 Mar 9;23(7):e13443. doi: 10.1111/obr.13443 (PMC9285539; doi:10.1111/obr.13443)
Supplement: Supplementary file 1 — Table S1: Details of search Table S2: Search history Table S3: Rationale for meta‐analysis inclusion and data processing Figure S4: Trim and fill analysis Figure S5: Sensitivity analysis of meta‐analysis excluding Leonard study (high risk): Forest plot showing standardised mean difference in taste preference of HFSS products between HFSS packaging with and without a character HFSS advert, by character type Figure S6: Bias assessment for experimental studies [file OBR-23-0-s001.docx]

**Supplemental file**

**Table S1: Details of search**

Databases

- Medline (OVID)
- Cochrane Library
- Scopus
- PsycInfo (OVID)
- ProQuest (Central)- ASSIA
- Web of Science- Social sciences and emerging sources citation indexes
- Social Policy and Practice (OVID)

**Inclusion criteria/search terms**

| **Participants** | 0-15 years | child* OR youth* OR adolescen* OR young people OR young person* OR schoolchild* OR boy* OR girl* OR teen* OR school child* NOT adult* |
| --- | --- | --- |
| **Exposure (intervention)** | Exposure to advertising of HFSS/unhealthy food advertisement using brand equity/licensed/mascot characters  Exposure to packaging | advert* OR market* OR commercial* OR promot* OR pack* OR label*, OR sticker* OR bag* OR box* OR tin* OR jar* OR carton* OR sleeve*, OR advergam* OR tag* OR lid* OR container* |
| **Exposure (brand characters)** | Exposure to brand equity/license/mascot characters within advertisement | brand* OR equity* OR character* OR brand character* OR brand equity* OR licens* OR brand licens* OR mascot* OR brand mascot* OR character licens* OR figure* OR brand figure* OR character figure* OR equity character* OR cartoon* |
| **Exposure (food)** | Intervention vs. no intervention  Comparison of HFSS food advertisement with characters and non-food advertisement control OR  HFSS food advertisement with character compared to HFSS food advertisement without character  Could also include a healthy food comparison group with OR without character. | Food* OR beverage* OR soda* OR cola* OR fizzy adj drink* OR carbonated adj beverage* OR carbonated adj drink* OR soft adj beverage* OR soft adj drink* OR high salt* OR high fat* OR high sugar* OR snack* OR sweet* OR meal* OR HFSS OR snack* OR sweet* OR meal* NOT alcohol* |
| **Outcome measure** | Any of these:  Measured energy intake (calories, grams or other units)/quantity of product consumed  Ad libitum consumption of food  Dietary behaviour  Preference of food  Like/dislike ratings  Purchase request  Purchase intentions | intake* OR consumption* OR food choice OR eating behavio* OR dietary intake OR energy consumption OR energy intake OR judgement* OR preference* OR liking OR favour* OR attitude OR purchas* OR buy* OR spend* OR request* OR pester* OR intent* |
| **Study designs** | Experimental; intervention (with or without a control group); real world studies |  |
| **Other**  Geography  Languages  Time | All  All  2009-2021 |  |

**Table S2: Search history**

| Medline (OVID)  1 exp adolescent/ or exp child/ or exp infant/  2 (child* or youth* or adolescen* young people or Young person* or boy* or girl* or teen* or Infant* or preschool* or toddler* or minor* or Babies or Baby).ti,ab,id.  3 1 or 2  4 Marketing/ or Advertising as topic/ or Product packaging/ or Food packaging/ or Food labelling/ or Advertisement/ or Social marketing/ or social media/  5 ((advert* adj2 food) or (advert* adj2 supermarket) or (advert* adj2 buy*) or (market* adj2 food) or (market* adj2 supermarket) or (market* adj2 buy*) or (promot* adj2 food) or (promot* adj2 supermarket) or (promot* adj2 buy*) or (promot* adj2 food) or (promot* adj2 supermarket) or (promot* adj2 buy*) or commercials or "TV commercial*" or "Television commercial*" or "radio commercial*" or "media commercial*" or digital adver* or digital market* or Mobile app* or Mobile application* or advergam* or Facebook or Instagram or Twitter or Snapchat or Youtube or Weibo or Wechat or Youku).ti,ab,id.  6 4 or 5  7 Food/ or Beverages/ or Carbonated beverages/  8 (food* or beverage* or Cola or Colas or Soda or sodas or (fizzy adj2 drink*) or (carbonated adj2 drink*) or (soft adj drink*) or high salt* or high fat* or high sugar* or snack* or sweet* or Meal* or HFSS).ti,ab,id.  9 7 or 8  10 exp Cartoons as Topic/  11 (brand* or equity* or character* or licens* or mascot* or figure* or cartoon*).ti,ab,id. 3398610  12 10 or 11  13 Eating/ or Snacks/ or Drinking/ or Food Preferences/ or Feeding Behavior/ or Diet, High-Fat/ or Diet/ or Diet, Western/ or energy intake/  14 (intake* or calori* or consumption* or food choice* or eating behavio* or snacking behavio* or preference* or favour* or favor* or purchas* or buy* or spend* or pester* or request* or liking).ti,ab,id.  15 13 or 14  16 3 and 6 and 9 and 12 and 15  17 16  18 limit 17 to yr="2009 -Current" |
| --- |
| Social Policy and Practice  1 (child* or youth* or adolescen* young people or Young person* or boy* or girl* or teen* or Infant* or preschool* or toddler* or minor* or Babies or Baby).ab,de,hw,nt,ti.  2 (advert* or (market* adj2 food) or (market* adj2 supermarket) or (market* adj2 buy*) or (promot* adj2 food) or (promot* adj2 supermarket) or (promot* adj2 buy*) or (promot* adj2 food) or (promot* adj2 supermarket) or (promot* adj2 buy*) or commercials or "TV commercial*" or "Television commercial*" or "radio commercial*" or "media commercial*" or digital adver* or digital market* or Mobile app* or Mobile application* or advergam* or Facebook or Instagram or Twitter or Snapchat or Youtube or Weibo or Wechat or Youku).ab,de,hw,nt,ti.  3 (food* or beverage* or Cola or Colas or Soda or sodas or (fizzy adj2 drink*) or (carbonated adj2 drink*) or (soft adj drink*) or high salt* or high fat* or high sugar* or snack* or sweet* or Meal* or HFSS).ab,de,hw,nt,ti.  4 (brand* or equity* or character* or licens* or mascot* or figure* or cartoon*).ab,de,hw,nt,ti.  5 (intake* or calori* or consumption* or food choice* or eating behavio* or snacking behavio* or preference* or favour* or favor* or purchas* or buy* or spend* or pester* or request* or liking).ab,de,hw,nt,ti.  6 1 and 2 and 3 and 4 and 5 |
| Web of Science  #1 TS= (child* OR youth* OR adolescen* young people OR Young person* OR boy* OR girl* OR teen* OR Infant* OR preschool* OR toddler* OR minor* OR Babies OR Baby)  Indexes=SSCI, ESCI  #2 TS=(advert* or (market* adj2 food) or (market* adj2 supermarket) or (market* adj2 buy*) or (promot* adj2 food) or (promot* adj2 supermarket) or (promot* adj2 buy*) or (promot* adj2 food) or (promot* adj2 supermarket) or (promot* adj2 buy*) or commercials or "TV commercial*" or "Television commercial*" or "radio commercial*" or "media commercial*" or digital adver* or digital market* or Mobile app* or Mobile application* or advergam* or Facebook or Instagram or Twitter or Snapchat or Youtube or Weibo or Wechat or Youku)  Indexes=SSCI, ESCI  #3 TS= (food* or beverage* or Cola or Colas or Soda or sodas or (fizzy adj2 drink*) or (carbonated adj2 drink*) or (soft adj drink*) or high salt* or high fat* or high sugar* or snack* or sweet* or Meal* or HFSS) Indexes=SSCI, ESCI  #4 TS=(brand* or equity* or character* or licens* or mascot* or figure* or cartoon*)  Indexes=SSCI, ESCI  #5 TS= (intake* or calori* or consumption* or food choice* or eating behavio* or snacking behavio* or preference* or favour* or favor* or purchas* or buy* or spend* or pester* or request* or liking)  Indexes=SSCI, ESCI  #6 ((((#5) AND #4) AND #3) AND #2) AND #1 Indexes=SSCI, ESCI |
| SCOPUS  1 TITLE-ABS-KEY ( child*  OR  youth*  OR  adolescen*  OR  "young people"  OR  "young person*"  OR  boy*  OR  girl*  OR  teen*  OR  infant*  OR  preschool*  OR  toddler*  OR  minor*  OR  babies  OR  baby )  2 TITLE-ABS-KEY ( advert*  OR  ( market*  W/2  food )  OR  ( market*  W/2  supermarket )  OR  ( market*  W/2  buy* )  OR  ( promot*  W/2  food )  OR  ( promot*  W/2  supermarket )  OR  ( promot*  W/2  buy* )  OR  commercials  OR  "TV commercial*"  OR  "Television commercial*"  OR  "radio commercial*"  OR  "media commercial*"  OR  ( digital  W/2  adver* )  OR  ( digital  W/2  market* )  OR  ( mobile  W/2  app* )  OR  ( mobile  W/2  application* )  OR  advergam  OR  facebook  OR  instagram  OR  twitter  OR  snapchat  OR  youtube  OR  weibo  OR  wechat  OR  youku )  3 TITLE-ABS-KEY ( food*  OR  beverage*  OR  cola  OR  colas  OR  soda  OR  sodas  OR  ( fizzy  W/2  drink* )  OR  ( carbonated  W/2  drink* )  OR  ( soft  W/2  drink* )  OR  "high salt*"  OR  "high fat*"  OR  "high sugar*"  OR  snack*  OR  sweet*  OR  meal*  OR  hfss )  4 TITLE-ABS-KEY ( brand*  OR  equity*  OR  character*  OR  licens*  OR  mascot*  OR  figure*  OR  cartoon* )  5 TITLE-ABS-KEY ( intake*  OR  calori*  OR  consumption*  OR  "food choice*"  OR  "eating behavio*"  OR  "snacking behavio*"  OR  preference*  OR  liking  OR  favor*  OR  favour*  OR  purchas*  OR  buy*  OR  spend*  OR  request*  OR  pester* )  6 ( TITLE-ABS-KEY ( ( child* OR youth* OR adolescen* OR "young people" OR "young person*" OR boy* OR girl* OR teen* OR infant* OR preschool* OR toddler* OR minor* OR babies OR baby ) ) ) AND ( TITLE-ABS-KEY ( ( advert* OR ( market* W/2 food ) OR ( market* W/2 supermarket ) OR ( market* W/2 buy* ) OR ( promot* W/2 food ) OR ( promot* W/2 supermarket ) OR ( promot* W/2 buy* ) OR commercials OR "TV commercial*" OR "Television commercial*" OR "radio commercial*" OR "media commercial*" OR ( digital W/2 adver* ) OR ( digital W/2 market* ) OR ( mobile W/2 app* ) OR ( mobile W/2 application* OR advergam OR facebook OR instagram OR twitter OR snapchat OR youtube OR weibo OR wechat OR youku ) ) ) AND ( TITLE-ABS-KEY ( ( food* OR beverage* OR cola OR colas OR soda OR sodas OR ( fizzy W/2 drink* ) OR ( carbonated W/2 drink* ) OR ( soft W/2 drink* ) OR "high salt*" OR "high fat*" OR "high sugar*" OR snack* OR sweet* OR meal* OR hfss ) ) ) AND ( TITLE-ABS-KEY ( ( intake* OR calori* OR consumption* OR "food choice*" OR "eating behavio*" OR "snacking behavio*" OR preference* OR liking OR favor* OR favour* OR purchas* OR buy* OR spend* OR request* OR pester* ) ) ) AND ( TITLE-ABS-KEY ( ( brand* OR equity* OR character* OR licens* OR mascot* OR figure* OR cartoon* ) ) ) |
| PsycInfo  1 ("100" or "120" or "140" or "160" or "180" or "200").ag.  2 (child* OR youth* OR adolescen* young people OR Young person* OR boy* OR girl* OR teen* OR Infant* OR preschool* OR toddler* OR minor* OR Babies OR Baby).ti,ab,id.  3 1 or 2  4 exp Marketing/ or exp Advertising/ or exp digital marketing/ or exp social marketing/ or exp television advertising/ or commercials/ or exp media exposure/ or exp social media/  5 ((advert* or (market* adj2 food) or (market* adj2 supermarket) or (market* adj2 buy*) or (promot* adj2 food) or (promot* adj2 supermarket) or (promot* adj2 buy*) or commercials or "TV commercial*" or "Television commercial*" or "radio commercial*" or "media commercial*" or digital adver* or digital market* or mobile app* or mobile application* or advergam* or facebook or instagram or twitter or snapchat or youtube or weibo or yechat or youku).ti,ab,id.  6 4 or 5  7 Food/ or Fast food/ or exp "Beverages (Nonalcoholic)"/  8 (food* or beverage* or cola or colas or soda or sodas or (fizzy adj2 drink*) or (carbonated adj2 drink*) or (soft adj drink*) or high salt* or high fat* or high sugar* or snack or sweet* or meal or HFSS).ti,ab,id.  9 7 or 8  10 exp food intake/ or exp food preferences/ or exp "Rumination (Eating)"/ or exp eating behavior/ or exp consumer behavior/ or exp brand preferences/  11 (intake* or calori* or consumption* or food choice* or eating behavio* or snacking behavio* or preference* or "food preferences" or favour* or favor* or purchas* or "food purchas*" or buy* or spend* or pester* or request* or liking).ti,ab,id.  12 10 or 11  13 (brand* or equity* or character* or licens* or mascot* or figure* or cartoon*).ti,ab,id.  14 3 and 6 and 9 and 12 and 13  15 limit 14 to yr="2009 -Current" |
| Cochrane  ID    Search  #1    MeSH descriptor: [Child] explode all trees  #2    MeSH descriptor: [Infant] explode all trees  #3    MeSH descriptor: [Adolescent] explode all trees  #4    (child* or youth* or adolescen* or "young people" or "young person*" or boy* or girl* or teen* or infant* or preschool* or toddler* or minor* or babies or baby).ti,ab,id.  #5    MeSH descriptor: [Marketing] explode all trees  #6    MeSH descriptor: [Advertising as Topic] explode all trees  #7    MeSH descriptor: [Product Packaging] explode all trees  #8    MeSH descriptor: [Food Packaging] explode all trees  #9    MeSH descriptor: [Food Labeling] explode all trees  #10    MeSH descriptor: [Advertisement] explode all trees  #11    MeSH descriptor: [Social Marketing] explode all trees  #12    (advert* or (market* adj2 food) or (market* adj2 supermarket) or (market* adj2 buy*) or (promot* adj2 food) or (promot* adj2 supermarket) or (promot* adj2 buy*) or commercials or "TV commercial*" or "Television commercial*" or "radio commercial*" or "media commercial*" or digital adver* or digital market* or mobile app* or mobile application* or advergam* or facebook or instagram or twitter or snapchat or youtube or weibo or yechat or youku).ti,ab,id.  #13    MeSH descriptor: [Food] explode all trees  #14    MeSH descriptor: [Beverages] explode all trees  #15    MeSH descriptor: [Carbonated Beverages] explode all trees  #16    (food* or beverage* or cola or colas or soda or sodas or (fizzy adj2 drink*) or (carbonated adj2 drink*) or (soft adj drink*) or high salt* or high fat* or high sugar* or snack or sweet* or meal or HFSS).ti,ab,id.  #17    MeSH descriptor: [Cartoons as Topic] explode all trees  #18    (brand* or equity* or character* or licens* or mascot* or figure* or cartoon*).ti,ab,id.  #19    MeSH descriptor: [Eating] explode all trees  #20    MeSH descriptor: [Feeding Behavior] explode all trees  #21    MeSH descriptor: [Snacks] explode all trees  #22    MeSH descriptor: [Drinking] explode all trees  #23    MeSH descriptor: [Food Preferences] explode all trees  #24    MeSH descriptor: [Diet] explode all trees  #25    MeSH descriptor: [Diet, High-Fat] explode all trees  #26    MeSH descriptor: [Diet, Western] explode all trees  #27    MeSH descriptor: [Energy Intake] explode all trees  #28    (intake* or calori* or consumption* or food choice* or eating behavio* or snacking behavio* or preference* or favour* or favor* or purchas* or buy* or spend* or pester* or request* or liking).ti,ab,id  #29    #1 OR #2 OR #3 OR #4  #30    #5 OR #6 OR #7 OR #8 OR #9 OR #10 OR #11 OR #12  #31    #13 OR #14 OR #15 OR #16  #32    #17 OR #18  #33    #19 OR #20 OR #21 OR #22 OR #23 OR #24 OR #25 OR #26 OR #27 OR #28  #34    #29 AND #30 AND #31 AND #32 AND #33 with Cochrane Library publication date Between Jan 2009 and Dec 2021, in Cochrane Reviews, Cochrane Protocols, Trials, Clinical Answers, Editorials, Special collections |
| Proquest ASSIA  1 MAINSUBJECT.EXACT.EXPLODE("children") OR MAINSUBJECT.EXACT.EXPLODE("adolescents") OR MAINSUBJECT.EXACT.EXPLODE("infant")  2 TI,AB(child* OR youth* OR adolescen* young people OR Young person* OR boy* OR girl* OR teen* OR Infant* OR preschool* OR toddler* OR minor* OR Babies OR Baby)  3 1 OR 2  4 MAINSUBJECT.EXACT.EXPLODE("Advertising") OR MAINSUBJECT.EXACT.EXPLODE("Advertisements") OR MAINSUBJECT.EXACT.EXPLODE("Marketing") OR MAINSUBJECT.EXACT.EXPLODE("Packaging")  5 TI,AB(advert* or (market* adj2 food) or (market* adj2 supermarket) or (market* adj2 buy*) or (promot* adj2 food) or (promot* adj2 supermarket) or (promot* adj2 buy*) or (promot* adj2 food) or (promot* adj2 supermarket) or (promot* adj2 buy*) or commercials or "TV commercial*" or "Television commercial*" or "radio commercial*" or "media commercial*" or digital adver* or digital market* or Mobile app* or Mobile application* or advergam* or Facebook or Instagram or Twitter or Snapchat or Youtube or Weibo or Wechat or Youku)  6 4 OR 5  7 MAINSUBJECT.EXACT("Drinks") OR MAINSUBJECT.EXACT.EXPLODE("Food")  8 TI,AB(food* or beverage* or Cola or Colas or Soda or sodas or (fizzy adj2 drink*) or (carbonated adj2 drink*) or (soft adj drink*) or high salt* or high fat* or high sugar* or snack* or sweet* or Meal* or HFSS)  9 7 OR 8  10 MAINSUBJECT.EXACT("Eating behaviour") OR MAINSUBJECT.EXACT("Food consumption")  11 TI,AB(intake* or calori* or consumption* or food choice* or eating behavio* or snacking behavio* or preference* or favour* or favor* or purchas* or buy* or spend* or pester* or request* or liking)  12 10 OR 11  13 TI,AB(brand* or equity* or character* or licens* or mascot* or figure* or cartoon*)  14 3 and 6 and 9 and 12 and 13 |

**Table S3: Rationale for meta-analysis inclusion and data processing**

| Lapierre, 2011  Intervention = HFSS breakfast cereal with licensed character (Happy Feet- Mumble and Gloria), titled either ‘healthy bits’ or ‘sugar bits’  Comparison = same product with no character, titled either ‘healthy bits’ or ‘sugar bits’  Outcome = after tasting the product asked “How much did you like the taste of the cereal?” and responded using a 5-point smiley face rating scale (1, deep frown: really do not like; 5, big smile: really like).  Design = between-subjects. |
| --- |
| Letona, 2012  Intervention = HFSS products (potato chips, honey graham cracker) with licensed characters (SpongeBob SquarePants, the Pink Panther or El Chavo)  Comparison = the same products with no characters  Excluded condition in meta-analysis = taste preference for baby carrots (included in sensitivity analysis)  Outcome = after tasting the paired products participants were asked: ‘How much do you like the way this tastes? Do you love it, like it, it’s OK, you don’t like it, or you hate it?’ and responded using 5-point Likert smiley-face scale. The scale ranged from 5 (love it) to 1 (hate it).  Design = Within-subjects, random order. |
| Leonard, 2019 – Study 3  Intervention= HFSS product (cookies) with licensed character (Scooby-Doo)  Comparison= same product with no character  Outcome= after tasting at least one of the products (with/without LC) asked taste rating question “How much do you like the way that this food tastes?” (response options: “Hate It,” “Don’t Like It,” “It’s OK,” “Like It,” and “Love It.”) and wanting question “How much would you like to have this food for a snack?” “Don’t Want!,” (response options: “Not Really,” “It Would Be OK,” “Kind Of,” and “Want It!.”). Both 5-point scales used smiley faces and were averaged together.  Design = Between-subject, randomisation unclear |
| McGale 2016 – Study 1  Intervention = 3 HFSS products (Cheestrings, Pom-Bear Potato, Coco Pops Snack Bar) matched with their brand equity characters (Coco the Monkey, Cheestring character, Pom-Bear character)  Comparison =  the same products with no characters  Outcome = after tasting the paired products participants were asked: “Do you love it, like it, it’s OK, don’t like it, or hate it?” and responded using a 5-point smiley face Likert scale.  Design = Within-subjects, random order. |
| McGale 2016 – Study 2  Intervention = 3 HFSS products (Cheestrings, Pom-Bear Potato, Coco Pops Snack Bar with matched with the wrong brand equity characters  Comparison = the same products with no characters  Outcome = after tasting the paired products participants were asked: “Do you love it, like it, it’s OK, don’t like it, or hate it?” and responded using a 5-point smiley face Likert scale.  Design = Within-subjects, random order. |

**Figure S4: Trim and fill analysis**


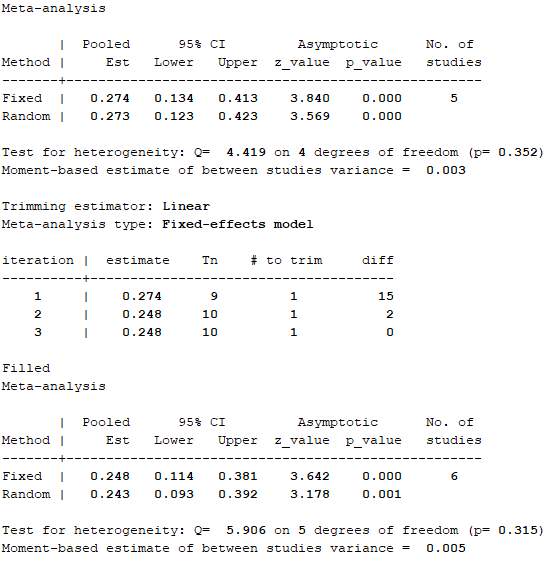


**Figure S5: Sensitivity analysis of meta-analysis excluding Leonard study (high risk): Forest plot showing standardised mean difference in taste preference of HFSS products between HFSS packaging with and without a character HFSS advert, by character type**


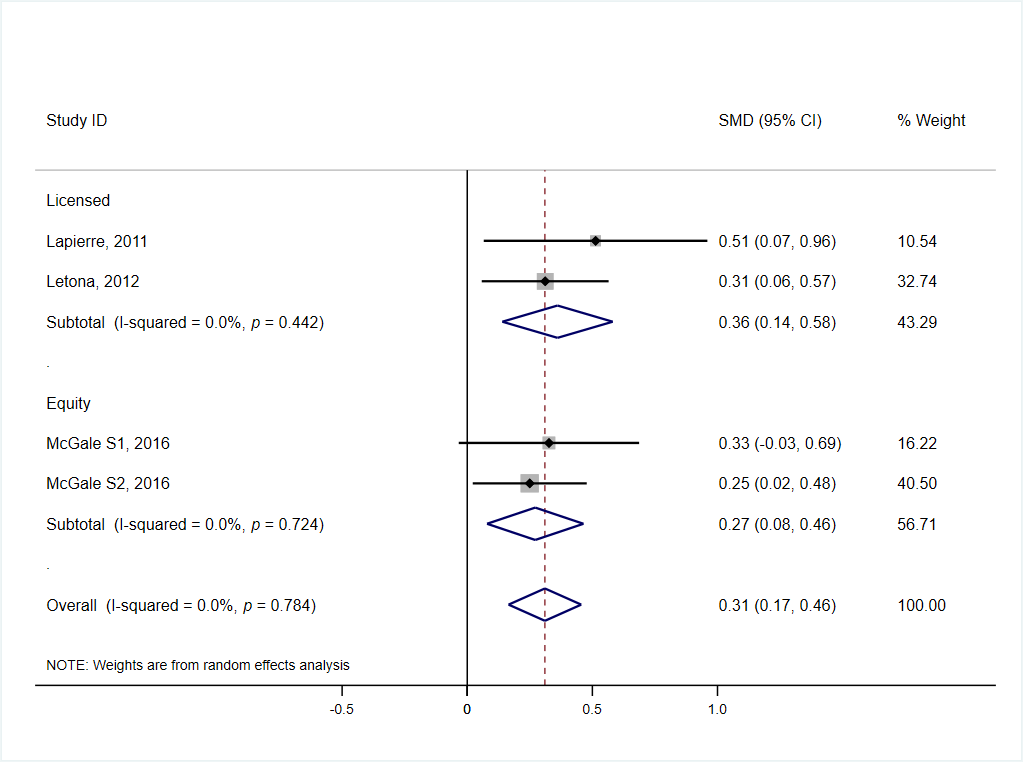


**Figure S6: Bias assessment for experimental studies**
